# Fusion primer driven racket PCR: A novel tool for genome walking

**DOI:** 10.3389/fgene.2022.969840

**Published:** 2022-10-18

**Authors:** Jinfeng Pei, Tianyi Sun, Lingqin Wang, Zhenkang Pan, Xinyue Guo, Haixing Li

**Affiliations:** ^1^ State Key Laboratory of Food Science and Technology, Nanchang University, Nanchang, China; ^2^ Sino-German Joint Research Institute, Nanchang University, Nanchang, China; ^3^ Key Laboratory of Poyang Lake Environment and Resource Utilization of Ministry of Education, School of Chemistry and Chemical Engineering, Nanchang University, Nanchang, China

**Keywords:** fusion primer, walking primer, partial annealing, racket-like DNA, genome-walking

## Abstract

The limitations of the current genome-walking strategies include strong background and cumbersome experimental processes. Herein, we report a genome-walking method, fusion primer-driven racket PCR (FPR-PCR), for the reliable retrieval of unknown flanking DNA sequences. Four sequence-specific primers (SSP1, SSP2, SSP3, and SSP4) were sequentially selected from known DNA (5'→3′) to perform FPR-PCR. SSP3 is the fragment that mediates intra-strand annealing (FISA). The FISA fragment is attached to the 5′ end of SSP1, generating a fusion primer. FPR-PCR comprises two rounds of amplification reactions. The single-fusion primary FPR-PCR begins with the selective synthesis of the target first strand, then allows the primer to partially anneal to some place(s) on the unknown region of this strand, producing the target second strand. Afterward, a new first strand is synthesized using the second strand as the template. The 3′ end of this new first strand undergoes intra-strand annealing to the FISA site, followed by the formation of a racket-like DNA by a loop-back extension. This racket-like DNA is exponentially amplified in the secondary FPR-PCR performed using SSP2 and SSP4. We validated this FPR-PCR method by identifying the unknown flanks of *Lactobacillus brevis* CD0817 glutamic acid decarboxylase genes and the rice hygromycin gene.

## Introduction

Genome-walking is a method that starts from a known region of the genome or genome library and then sequentially mines the unknown flanking sequences (Leoni et al., 2008; Kotik, 2009). Genome-walking has practical significance in molecular biology and related fields. The successful applications of genome-walking include 1) obtaining the regulatory genes of structural genes; 2) amplifying non-conserved sequences based on the conserved regions of genes; 3) identifying insertion sites for T-DNA or transposons; 4) filling gaps in whole-genome sequencing; 5) discovering new functional genes; and 6) screening microbes (Myrick and Gelbart, 2007; Kotik, 2009; Thirulogachandar et al., 2011; Fraiture et al., 2021; Zhou et al., 2021).

Genome-walking generally includes genome library and PCR-based strategies. The former strategy is cumbersome and labor-intensive due to the construction and then screening of genomic DNA libraries (Li et al., 2015; Zeng et al., 2020). The PCR-based strategy is attractive because of its efficiency and rapidity (Kotik, 2009; Alquezar-Planas et al., 2020). Several PCR-based genome-walking methods have been developed and validated for identifying unknown flanking DNA sequences. These PCR-based methods vary substantially in experimental processes but can be divided into four categories: 1) inverse PCR (Ochman et al., 1988; Uchiyama and Watanabe, 2006); 2) panhandle PCR; 3) ligation-dependent PCR (Yan et al., 2003; Yik et al., 2021); and 4) randomly primed PCR (Sun et al., 2022; Tan et al., 2005; Zhang et al., 2018).

Inverse PCR was the first described PCR-based genome-walking method. In this method, the genomic DNA is digested by an endonuclease. The digested fragments then undergo self-circularization with the help of DNA ligase and the circularized DNA is amplified, like classical end-to-end PCR, by a sequence-specific primer (SSP) pair. The two primers are in the orientation of the 5′ end facing the 5′ end, opposite to the classical one of the 3′ end facing the 3′ end (Ochman et al., 1988; Triglia et al., 1988). Inverse PCR has high specificity owing to the use of an SSP pair. However, the efficiency of this method is hindered by the complicated steps before PCR and the preferential amplification of small DNA (Kotik, 2009; Chang et al., 2018).

Panhandle PCR, as the name implies, requires that the target DNA form a panhandle-like structure before PCR. In routine panhandle PCR, the formation of panhandle-like DNA relies on the digestion of genomic DNA. The ends of the digested product are then ligated to a fragment homologous to a known site (Jones and Winistorfer, 1992; Jones and Winistorfer, 1997). The two improved versions, universal fast walking (Myrick and Gelbart, 2002) and self-formed adapter (Wang et al., 2007) PCR, use a walking primer (with a 5′ part homologous to known DNA) to mediate the formation of panhandle-like DNA through a random priming strategy. Universal fast walking provides long-range walking because only the most distal site partially bound to the primer is further processed as follows: exonuclease I digestion stops at the branch point of this site; thereafter, DNA polymerization starts from the trimmed target DNA 3′ end using the 5′ part of the primer as the template. The extended 3′ end can mediate the formation of panhandle-like DNA (Myrick and Gelbart, 2002). A panhandle PCR is completed using SSP pairs; thus, it shows equivalent amplification specificity to that of inverse PCR.

Like inverse PCR, ligation-dependent PCR also requires endonuclease digestion. The digested products are then ligated to random DNA. Subsequently, the target DNA is enriched by 2–3 rounds of PCR in which the random primers sequentially pair with nested SSPs (Yan et al., 2003; Ji and Braam, 2010). However, background arising from the random primer alone is a drawback (Kotik, 2009; Li et al., 2015).

Randomly primed PCR does not involve any extra operation before PCR (Jia et al., 2017). In the primary PCR, a relaxed-stringency cycle is required to facilitate the walking primer to partially anneal to the unknown flank to create a DNA of interest. This DNA is then gradually enriched by 2–3 rounds of nested PCR based on the superior annealing efficiency of SSPs to the walking primer (Liu and Whittier, 1995; Chang et al., 2018). Like ligation-dependent PCR, the reliability of randomly primed PCR is reduced by the non-target amplification of the walking primer (Kotik, 2009; Yu et al., 2020).

Herein, we propose a novel PCR-based genome-walking method, fusion primer-driven racket PCR (FPR-PCR). This method uses a tri-functional primer fused by two sequence-specific fragments formed in the primary PCR. The tri-functional primer mediates walking, selective enrichment, and intra-strand annealing of the target DNA. We verified the practicability of this method by amplifying the unknown flanks of the *Lactobacillus brevis* CD0817 glutamic acid decarboxylase genes (*gadR/C*) (Gao et al., 2019; Jia et al., 2022) and the rice hygromycin gene (*hyg*) (Li et al., 2015).

## Materials and methods

### Genomic DNA isolation

The genomic DNA of *L. brevis* CD0817 (Gao et al., 2019) was isolated using the TIANamp Bacteria DNA Kit (TIANGEN Biotech Co., Ltd., Beijing, China) according to the manufacturer’s instructions.

### Primer design

The genome (CP032931.1) of *L. brevis* CD0817 and the *hyg* gene (KF206149.1) (Gao et al., 2019; Li et al., 2015, 2022) with the surrounding regions are available in GenBank. A part of *gadR* (AYM03984.1), *gadC* (AYM03983.1), or *hyg* was chosen as the “known sequence” for SSP design, while the adjacent region was assumed to be “unknown sequence”. The “known sequence” plus its “unknown sequence” is here collectively called the “reference sequence” (Supplementary Figure S1). In this study, five sequence-specific fragments with the same orientation, namely, two SSP1s (SSP1α and SSP1β), one SSP2, one SSP3 (fragment mediating intra-strand annealing [FISA]), and one SSP4, were sequentially chosen in the direction of 5′ to 3′ from each known region. Two fusion primers (FPα and FPβ) were made by attaching the FISA to the 5′ ends of SSP1α and SSP1β, respectively, to perform two parallel FPR-PCRs. SSP1α and SSP1β can be entirely irrelevant or partially overlapping; in the latter case, the difference between the two fragments’ 3′ ends must be at least 3 nt. The FISA is 17–18 nt with a Tm of 45–55°C. The SSP1s are 17–21 nt with a Tm of 50–55°C. The fusion primers are 34–39 nt with a Tm of 65–70°C. SSP2 or SSP3 are 25–30 nt with a Tm of 60–66°C. Any primer or primer pair must be free of severe self-hairpin or dimer. The primers used in the present study are listed in Table 1.

**TABLE 1:** Primers used in this study.

Fusion primer used in primary PCR			SSP2/SSP4 primer pair used in secondary PCR	
Fusion primer	FISA	SSP1		
*gadR*-FPα	CGTAAACCTGCGTAAAAA	GTC​CAT​ACC​CTC​ATC​TCC​ATT	*gadR-*SSP2	AAC​TAT​CAC​CCC​ACA​ACG​TCA​TCT​C
	(233–250)	(38–57)		(194–218)
*gadR*-FPβ	CGTAAACCTGCGTAAAAA	AAT​GTC​CTT​CGT​TCT​TGA​T	*gadR-*SSP4	ACC​GTT​CAT​AGG​CGA​AAT​TGT​TTG​T
	(233–250)	(19–37)		(372–396)
*gadC*-FPα	TGTTTTCTTCTTGCTCT	ATGGTTATTCTCTGGGG	*gadC*-SSP2	TTG​GGC​GTT​ATA​ATT​CCT​GTT​TTC​TTC​TTG
	(132–148)	(34–50)		(115–144)
*gadC*-FPβ	TGTTTTCTTCTTGCTCT	TCTCTGGGGATTGATTG	*gadC*-SSP4	GGA​GCG​GTA​GTG​TGT​TAG​TTG​GGT​T
	(132–148)	(42–58)		(227–251)
*hyg*-FPα	GGACCGATGGCTGTGTA	TGGTTGGCTTGTATGGA	*hyg*-SSP2	CGG​GAC​TGT​CGG​GCG​TAC​ACA​AAT​C
	(321–337)	(83–99)		(274–298)
*hyg*-FPβ	GGACCGATGGCTGTGTA	TCATTGACTGGAGCGAG	*hyg*-SSP4	GAC​CGA​TGG​CTG​TGT​AGA​AGT​ACT​C
	(321–337)	(12–28)		(322–346)

Note: Fusion primer was made by attaching FISA (underlined) to SSP1 fragment 5′ end in the same row. SSP, sequence-specific primer; FISA, fragment mediating intra-strand annealing; FP, fusion primer; *gad*, l-glutamic acid decarboxylase gene; *hyg*, hygromycin gene. The first nucleotide of the known sequence (5’→3′) is numbered 1.

### PCR procedure

Each FPR-PCR comprised two rounds of PCR amplification. The primary PCR was conducted using a single fusion primer with genomic DNA as the template. The secondary PCR used the primary product as the template and included SSP2 and SSP4. To ensure walking success and efficiency, two parallel FPR-PCRs were simultaneously performed for any selected gene locus. The two primary PCRs were individually driven by the two fusion primers, while the two secondary PCRs were driven by the universal SSP primers SSP2 and SSP4.

The 50-μL primary reaction mixture included 1×LA PCR buffer II (Mg^2+^ Plus), 0.4 mM of each dNTP, genomic DNA (10–100 ng for the microbe and 100–1,000 ng for rice), 0.2 μM of a fusion primer, and 2.5 U of TaKaRa LA Taq polymerase (TaKaRa, Perking, China). The 50-μL secondary reaction mixtures included 1×LA PCR buffer II (Mg^2+^ plus), 0.4 mM of each dNTP, 1µL/50 µL primary PCR product, 0.2 μM of SSP2, 0.2 μM of SSP4, and 2.5 U of TaKaRa LA Taq polymerase.

The primary PCR comprised three annealing stages: stage 1, five moderate-stringency (55°C) cycles; stage 2, one low-stringency (25°C) cycle; and stage 3, thirty high-stringency (65°C) cycles. The secondary PCR consisted of twenty-five moderate high-stringency (60°C) cycles. The detailed thermal cycling conditions for FPR-PCR are presented in Table 2. The PCR reactions were performed on a Biometra TOne 96G PCR thermal cycler (Analytik Jena, Germany).

**TABLE 2:** Thermal cycling parameters for FPR-PCR.

Round of PCR	Thermal conditions	Cycle number
Primary	94°C 2 min	
	94°C 30 s, 55°C 30 s, 72°C 3 min	5
	94°C 30 s, 25°C 30 s, 72°C 3 min	1
	94°C 30 s, 65°C 30 s, 72°C 3 min	30
	72°C 10 min	
Secondary	94°C 2 min	
	94°C 30 s, 60°C 30 s, 72°C3 min	25
	72°C 10 min	

### DNA manipulation and sequencing

The PCR products were visualized by electrophoresis on 1.5% agarose gels. The major bands of the secondary PCRs were purified with the DiaSpin column DNA Gel Extraction Kit (Sangon Biotech Co. Ltd., Shanghai, China) and then sent to Sangon Biotech Co., Ltd. for sequencing using SSP2 and SSP4. This company uses ABI sequencers (3730xl DNA Analyzer) for DNA sequence analysis.

## Results

### FPR-PCR outline

The four sequence-specific primers (SSP1, SSP2, SSP3 [FISA], and SSP4) used in FPR-PCR are successively located on the known DNA in the 5′ to 3′ direction. The FPR-PCR comprises two rounds (primary and secondary) of nested amplification reactions. The SSP3 (FISA) is attached to the 5′ end of SSP1 to make a fusion primer mediating the primary FPR-PCR. SSP2 and SSP4 are used together in the secondary FPR-PCR.

The primary FPR-PCR starts with five moderate-stringency (55°C) cycles that permit the fusion primer 3′-part (SSP1) to hybridize only to its complement in the known region to increase the copies of the target first-strand. The subsequent single low-stringency (25°C) cycle helps the fusion primer to partially anneal to place(s) in the unknown region of this strand and extend toward the known region to produce the target second-strand, encompassing the fusion primer and its inverted repeat. Next, the target DNA is exponentially amplified in the following high-stringency (65°C) cycles. Some first-strands undergo intra-strand annealing between the FISA and its inverted repeat at the 3′ end; thereafter, the recessed 3′ end extends along the protruding known region. As a result, a racket-like DNA is formed, in which the unknown single-stranded region is defined by the known double-stranded stem. The secondary PCR is classical end-to-end PCR driven by SSP2 and SSP4 to amplify the target DNA exponentially. Any non-target DNAs cannot be amplified due to the lack of a perfect binding site to SSP2 or SSP4. Finally, the target DNA becomes the major product.

More than one fragment may be chosen near the SSP1 site. A corresponding number of fusion primers can then be created by appending the FISA to the 5′ ends of the chosen fragments. In this situation, parallel FPR-PCRs can be performed to guarantee genome-walking success and efficiency. The present study used two SSP1 fragments (SSP1α and SSP1β) from each given gene, thus resulting in two parallel sets of FPR-PCRs.

### FPR-PCR validation

**FIGURE 1:**
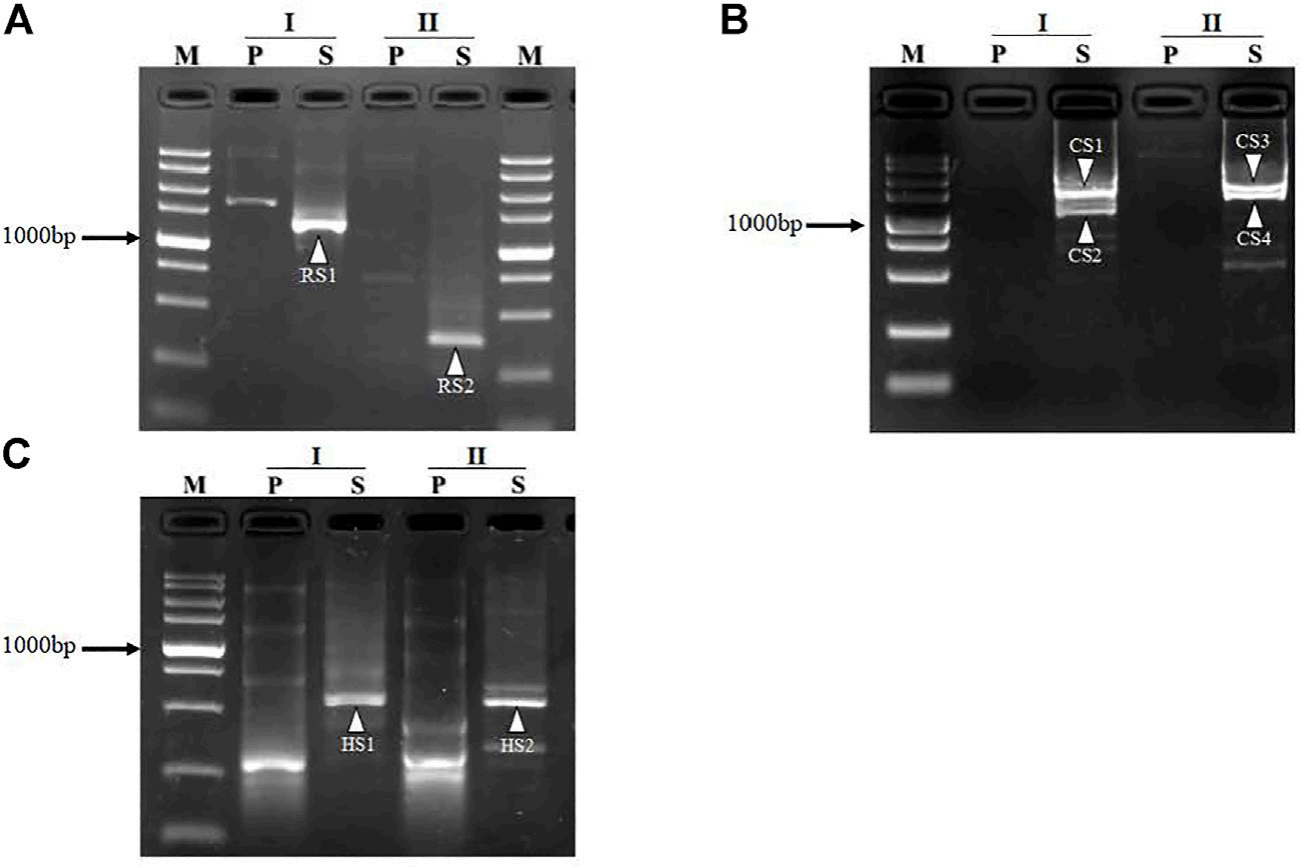
Mining unknown regions flanking *gadR* **(A)** and *gadC* **(B)** in *Lactobacillus brevis* CD0817 and *hyg* **(C)** in rice. I and II denote the two sets of FPR-PCRs in each round of walking. The bands RS1-RS2, CS1-CS4, and HS1-HS2, indicated by white arrowheads, are the secondary PCR products for *gadR*, *gadC*, and *hyg*, respectively. Lane P, primary PCR; lane S, secondary PCR; lane M, TaKaRa DL5000 DNA marker (from top to bottom 5000, 3000, 2000, 1,500, 1,000, 750, 500, 250, and 100 bp).

To validate the practicability of FPR-PCR, we applied the method to retrieve the unknown sequences for the segments adjacent to *L. brevis* CD0817 *gadR/C* genes and the rice *hyg* gene. We designed two fusion primers (FPα and FPβ) and one nested SSP primer pair SSP2/SSP4 (Table 1) for each gene to perform FPR-PCR. Thus, each walk in this study comprised two parallel sets of FPR-PCRs. The two primary PCRs were individually performed by the two fusion primers, while the two secondary PCRs were performed by the universal nested SSP primer pair. The detailed experimental processes are described in the Materials and Methods section. After walking, the PCR products were subjected to electrophoretic analysis on 1.5% agarose gels. As shown in Figure 1, each secondary FPR-PCR exhibited 1 – 2 clear DNA band(s). The lengths of the mined unknown DNAs ranged from 0.3 to 1.6 kb in the three walks, with the longest walking distances of 1.2, 1.6, and 0.5 kb for *gadR*, *gadC*, and *hyg*, respectively. These DNA bands were recovered and directly sequenced. The sequencing data showed that all the DNAs were of interest, as each overlapped with the end of the corresponding known region. These results validated the efficiency of the proposed FPR-PCR method.

### Analysis of random annealing sites of fusion primers

**FIGURE 2:**
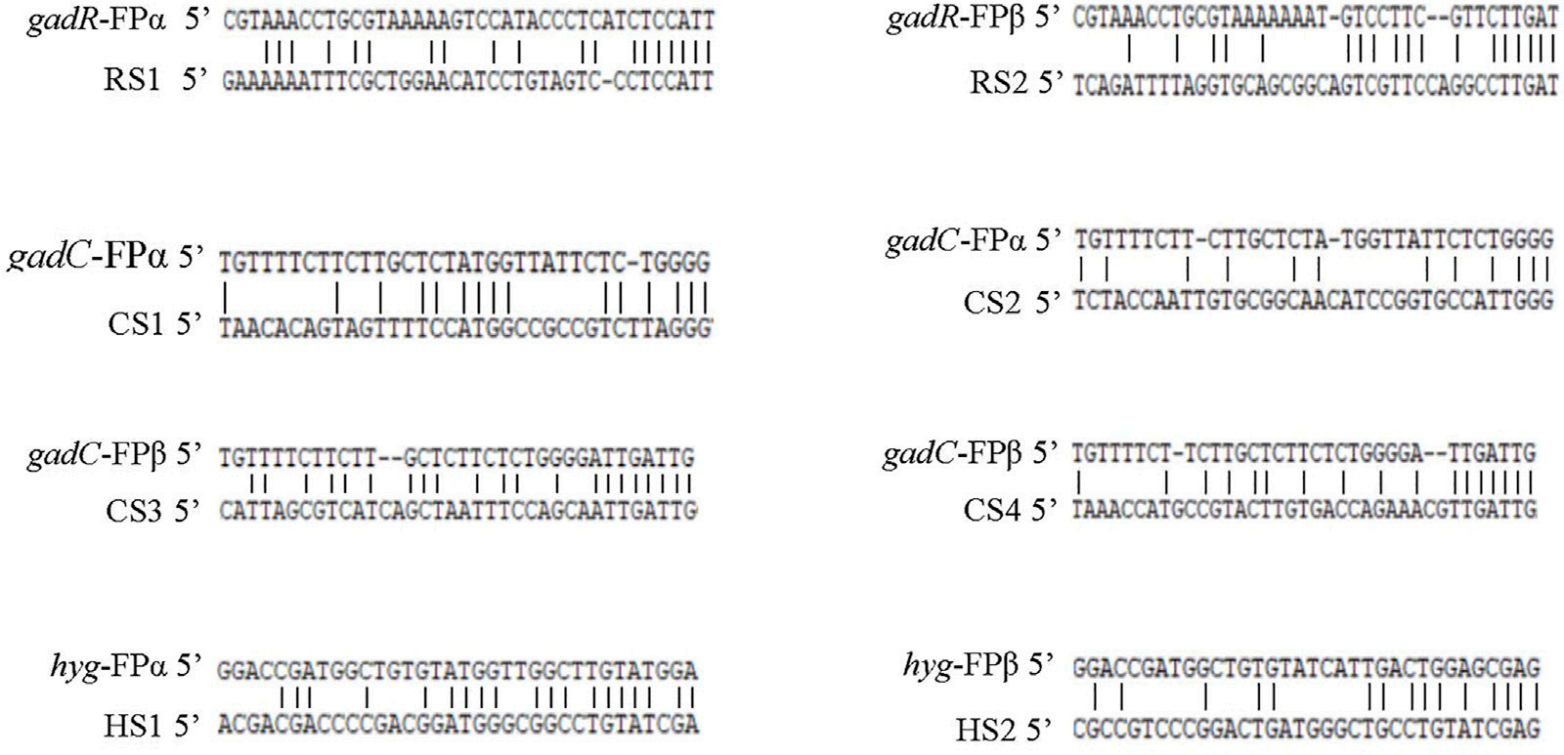
Partial annealing loci of the fusion primers. The upper and lower sequences are the fusion primer and annealing site, respectively. RS1-RS2, CS1-CS4, and HS1-HS2 correspond to the DNA bands described in Figure 1.

Based on the sequencing data of the obtained target DNA bands RS1-RS2, CS1-CS4, and HS1-HS2 (Figure 1), sequence alignments between the fusion primers and their partial annealing loci were performed, as summarized in Figure 2. The results demonstrated that any functional partial annealing showed a correct match of at least two base pairs at the 3′ terminus of a fusion primer. We also observed that the total number of matched base pairs ranged from 14 to 19. In general, the base paring was skewed towards primer 3′ end.

## Discussion

The currently available PCR-based genome-walking methods are unsatisfactory due to limitations including low success rates, high background, and complicated procedures (Myrick and Gelbart, 2002; Wang et al., 2022). Therefore, practical PCR-based genome-walking methods have been rigorously pursued. In this study, we report an efficient and reliable genome-walking method, FPR-PCR. The key to this method is the use of a tri-functional primer in primary PCR, which is created by splicing FISA to the 5′ end of SSP1. The three functions of this primer are: (I) mediate genomic walking by acting as the walking primer; (II) mediate sequence-specific annealing through the 3′ end of SSP1 to enhance the specificity; and (III) mediate intra-strand annealing through the FISA sequence to integrate known sequence into another site in the unknown region.

The primary amplification in existing randomly primed PCR methods requires a walking primer with a random sequence, in addition to the outermost SSP. Therefore, three types of undesired species are generally formed, including (I) SSP alone; (II) the combination of SSP and walking primer; and (III) the walking primer alone (Liu and Whittier, 1995; Kotik, 2009; Wang et al., 2022). In the FPR-PCR primary reaction, however, the fusion primer acts as not only SSP but also the walking primer. Thus, only one type of non-target species, primed by this fusion primer alone, is produced. This feature of FPR-PCR contributes to the specificity of this method.

The secondary FPR-PCR is completely sequence-specific. In this regard, FPR-PCR has identical specificity as those of the inverse (Ochman et al., 1988; Triglia et al., 1988) or panhandle (Jones, 1995; Myrick and Gelbart, 2002; Wang et al., 2007) PCR methods. However, inverse PCR requires DNA digestion and ligation before amplification, while panhandle PCR requires the complicated step to form the panhandle-like DNA. Compared to these strategies, the proposed FPR-PCR is a highly intensive and streamlined method involving two seamlessly connected, single-tube PCRs and a regular thermal cycling program. From the aspect of convenience, FPR-PCR is superior to both inverse and panhandle PCR methods.

The annealing temperature of the low-stringency cycle in primary FPR-PCR is low as 25°C. At such low temperatures, the fusion primer may identify some site(s) on the unknown flank suitable for partial annealing. Once this partial annealing occurs, walking should succeed. This low-stringency cycle is crucial to the success of FPR-PCR. Additionally, the ability to perform parallel simultaneous experiments provides a further guarantee for the success of FPR-PCR, as at least one FPR-PCR will give a positive outcome. FPR-PCR provides a high success rate similar to those reported for randomly primed PCRs, such as partially overlapping primer-based PCR (Li et al., 2015), stepwise partially overlapping primer-based PCR (Chang et al., 2018), and wristwatch PCR (Wang et al., 2022).

A straightforward genome-walking method has been highly pursued to allow rapid data acquisition and technology advances. FPR-PCR is one such straightforward genome-walking method, as it abolishes not only DNA pretreatments before PCR but also subsequent molecular cloning that are standard in the currently prevailing methods. Another criterion for straightforwardness met by FPR-PCR is that the purified secondary amplicon can be directly sequenced due to the sufficient amplification specificity.

**FIGURE 3:**
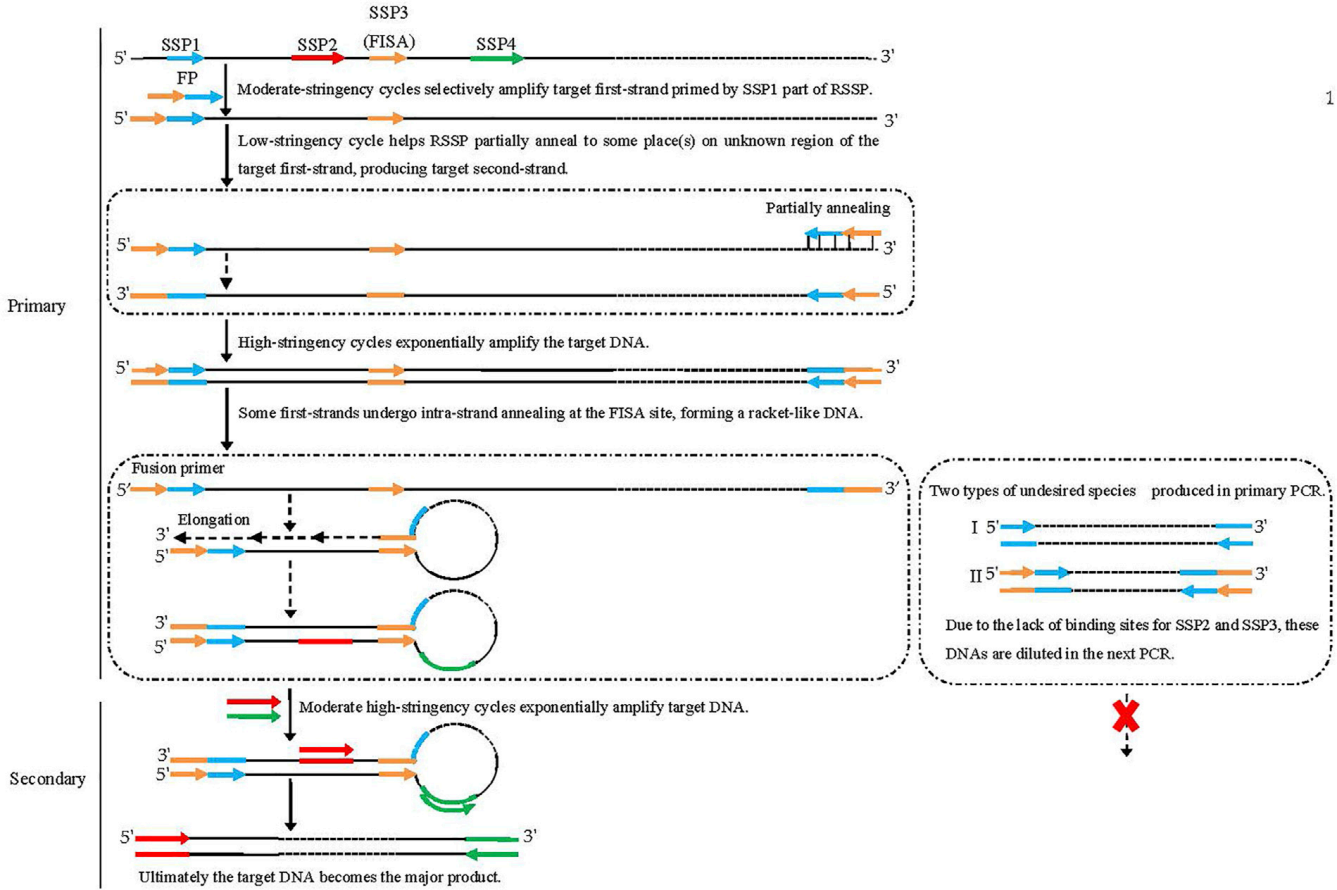
Schematic diagram of fusion primer-driven racket PCR. The fusion primer 3′-part is SSP1, while the 5′-part is the FISA fragment. Black solid lines: known sequences; black dashed lines: unknown sequences; colored solid lines with arrows: primers; colored solid lines: primers complements.

Most PCR-based methods have walking abilities of 0.3–3.0 kb (Liu and Whittier, 1995; Tan et al., 2005; Ashrafmansouri et al., 2020). However, universal fast walking (Myrick and Gelbart, 2002) and self-formed adapter (Wang et al., 2007) PCR show stronger walking abilities. The universal fast walking benefits from the fact that only the most distal site bound by walking primer can mediate intra-strand annealing (Myrick and Gelbart, 2002). Regarding self-formed adapter PCR (Wang et al., 2007), the improvement is presumably due to the limited number of templates, as weak partial annealing of the walking primer has few opportunities to form the adapter. Therefore, the probability of obtaining a long target DNA increases due to the major partial annealing. In the present study, the longest walking distances were 1.2, 1.6, and 0.5 kb, respectively, for the loci of *gadR*, *gadC*, and *hyg* (Figure 3). However, the length of the target amplicon is unpredictable in any PCR-based walking method, as it depends on the distance from the major partial annealing site of the walking primer to the known region. FPR-PCR can ensure walking efficiency by employing parallel experiments.

As discussed above, FPR-PCR is an effective genome-walking scheme based on its specificity, probability of success, walk length, and experimental process. Table 3 summarizes FPR-PCR and available PCR-based genome-walking methods from these important aspects.

**TABLE 3:** Comparison of PCR-based genome-walking approaches.

Approach	Rationale	Efficiency^a^				References
		Safeguard for specificity	Extra safeguard for success	Walk-length (kb)^b^	Operation before PCR	
Inverse PCR	Genomic DNA is self-circularized after digestion. The circularized DNA is amplified by two SSPs with opposite orientations	SSP pair	No	0.3–1.8	Digestion and ligation	Ochman et al., 1988; Triglia et al., 1988
Panhandle PCR	An oligo homologous to known region is appended to the end of unknown flank. Then panhandle-like DNA is formed under the mediation of this oligo. This DNA is amplified by SSP pair(s)	SSP pair	No	0.7–9.0	Digestion and ligation (universal fast walking only requires primer digest)	Jones and Winistorfer, (1992); Jones and Winistorfer, (1993); Jones and Winistorfer, (1997); Myrick and Gelbart, (2002); Myrick and Gelbart, (2007)
ligation-dependent PCR	Genomic DNA is digested then ligated with a random oligo. The ligated molecule is amplified by oligo primer and nested SSP	One-sided SSP	No	0.3–3	Digestion and ligation	Yan et al., 2003; Yik et al., 2021
Randomly primed PCR	A random primer anneals to unknown region in a low-stringency cycle, generating a pool of single-stranded DNAs. The target single-stranded DNA can be converted into double-stranded form by the SSP in the next high-stringency cycle; while non-target one cannot be further processed	One-sided SSP	Parallel experiments	0.3–3.5	No	Liu and Whittier, (1995); Wang et al., 2022
FPR-PCR	See in the section of *“Outline of FPR-PCR”*	SSP pair	Parallel experiments	0.5–1.6	No	This study

Remark: FPR-PCR combines the merits and addresses the limitations of existing PCR-based genome-walking methods. First, FPR-PCR overcomes non-target amplification from random walking primers. Second, FPR-PCR has equivalent convenience of traditional randomly primed PCR by avoiding steps before PCR. Third, FPR-PCR provides ensures success by executing parallel experiments. FPR-PCR has no advantage over the other methods in terms of walking ability. However, walking ability is variable and unpredictable in almost all PCR-based genome-walking methods. Taking overall efficiency into account, FPR-PCR should be considered as a prospective method

^a^ Specificity, success rate, walk length, and operational process all contribute to efficiency.

^b^ The walk length of universal fast walking depends on the performance of the DNA polymerase. FPR-PCR: fusion primer-driven racket PCR; SSP: sequence-specific primer.

In some situations, more than one clear target DNA band appeared (Figure 1). This multiple-band phenomenon is common in randomly primed PCRs and occurs due to the annealing of the walking primers to multiple sites on the unknown flank in the low-stringency cycle (Chang et al., 2018; Wang et al., 2022). At most two major bands appeared in each secondary FPR-PCR in the present study, much fewer than the approximately five bands reported in the other methods (Chang et al., 2018; Wang et al., 2022). As mentioned, the amplicons resulting from the primary FPR-PCR encompassed the fusion primer and its inverted repeat. These amplicons tend to form hairpins *via* intra-strand annealing mediated by the ends. In the secondary PCR, any minority product or short fragment derived from the primary PCR is preferentially formed into a hairpin instead of being amplified. The annoying multi-band phenomenon common in routine randomly primed PCR is, thus, significantly improved in FPR-PCR. This helps the PCR system to centralize limited resources to intensify the target product(s), improving the conciseness of the amplification result.

Amplification can occur even for oligonucleotide sequence mismatch rates of up to 60% between the walking primer and the annealing site (Parker et al., 1991). The match of the primer 3′ end is especially important for priming DNA synthesis (Parks et al., 1991). The pairing of two bases at the 3′ end of the walking primer appears to be mandatory for initiating primer extension (Parker et al., 1991; Parks et al., 1991). In the present study, the number of paired bases between fusion primers and the correspondingly partial annealing loci ranged from 14 to 19 bases, among which at least two were located at the 3′ end of the primer (Figure 1). Our results are similar to those reported previously (Parker et al., 1991; Parks et al., 1991).

## Conclusion

FPR-PCR, a genome-walking approach with high efficiency, was developed to retrieve unknown flanking DNA sequences. The feasibility of the proposed method was validated by probing the unknown flanks of the *gadR/C* genes in *L. brevis* CD0817 and the *hyg* gene in rice. Thus, FPR-PCR is a promising alternative to the available genome-walking protocols.

## Data Availability

This study analyzed publicly available datasets. These data can be found on GenBank under accession nos. CP032931.1 and KF206149.1.
