# Supplementary material for: Fusion primer driven racket PCR: A novel tool for genome walking
Source: Front Genet. 2022 Oct 18;13:969840. doi: 10.3389/fgene.2022.969840 (PMC9623105; doi:10.3389/fgene.2022.969840)
Supplement: Supplementary file 1 [file DataSheet1.PDF]

a

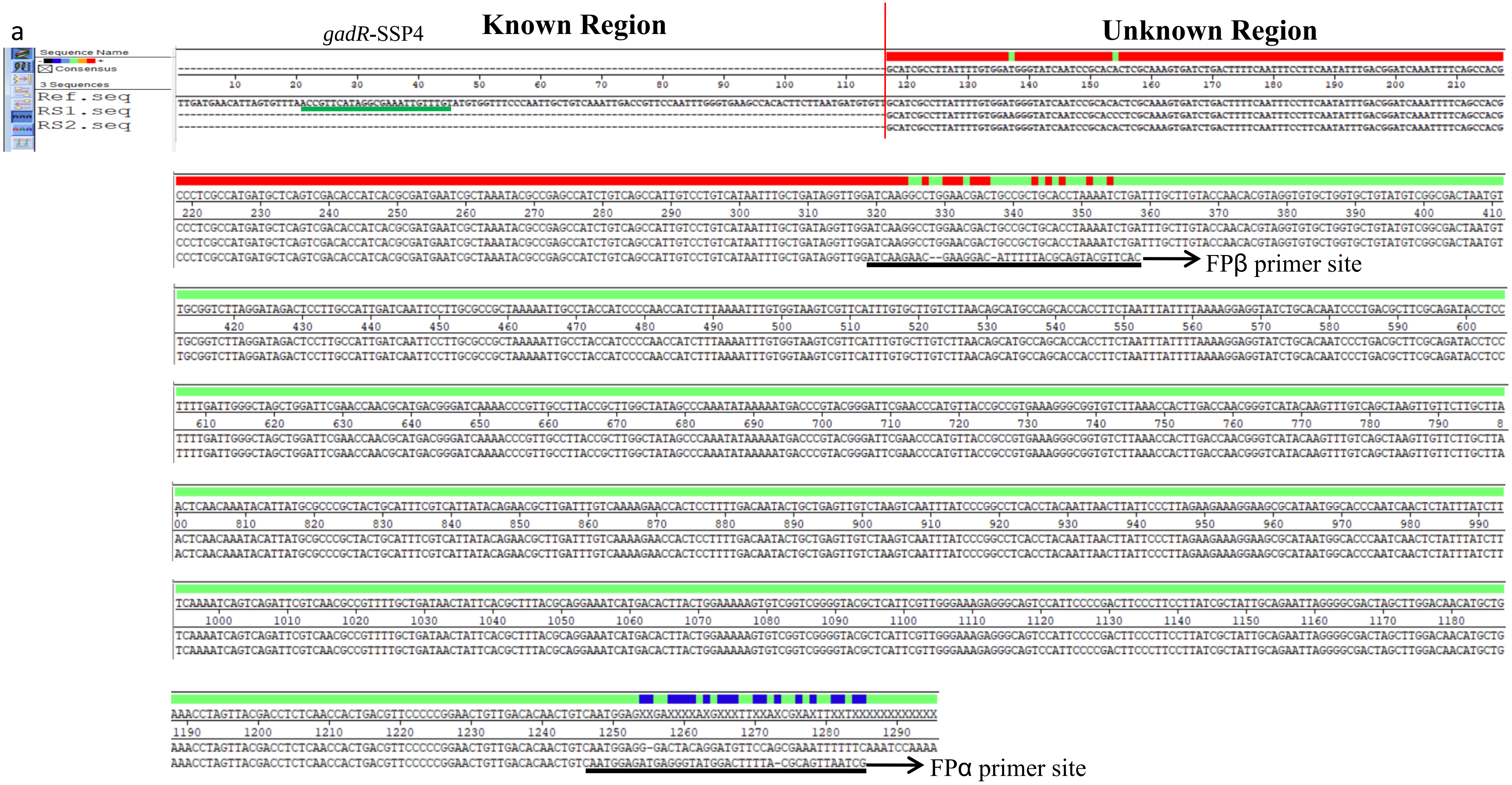

gadR-SSP4
Known Region
Unknown Region
Sequence Name
Consensus
3 Sequences
Ref.seq
RS1.seq
RS2.seq
FPβ primer site
FPα primer site

b

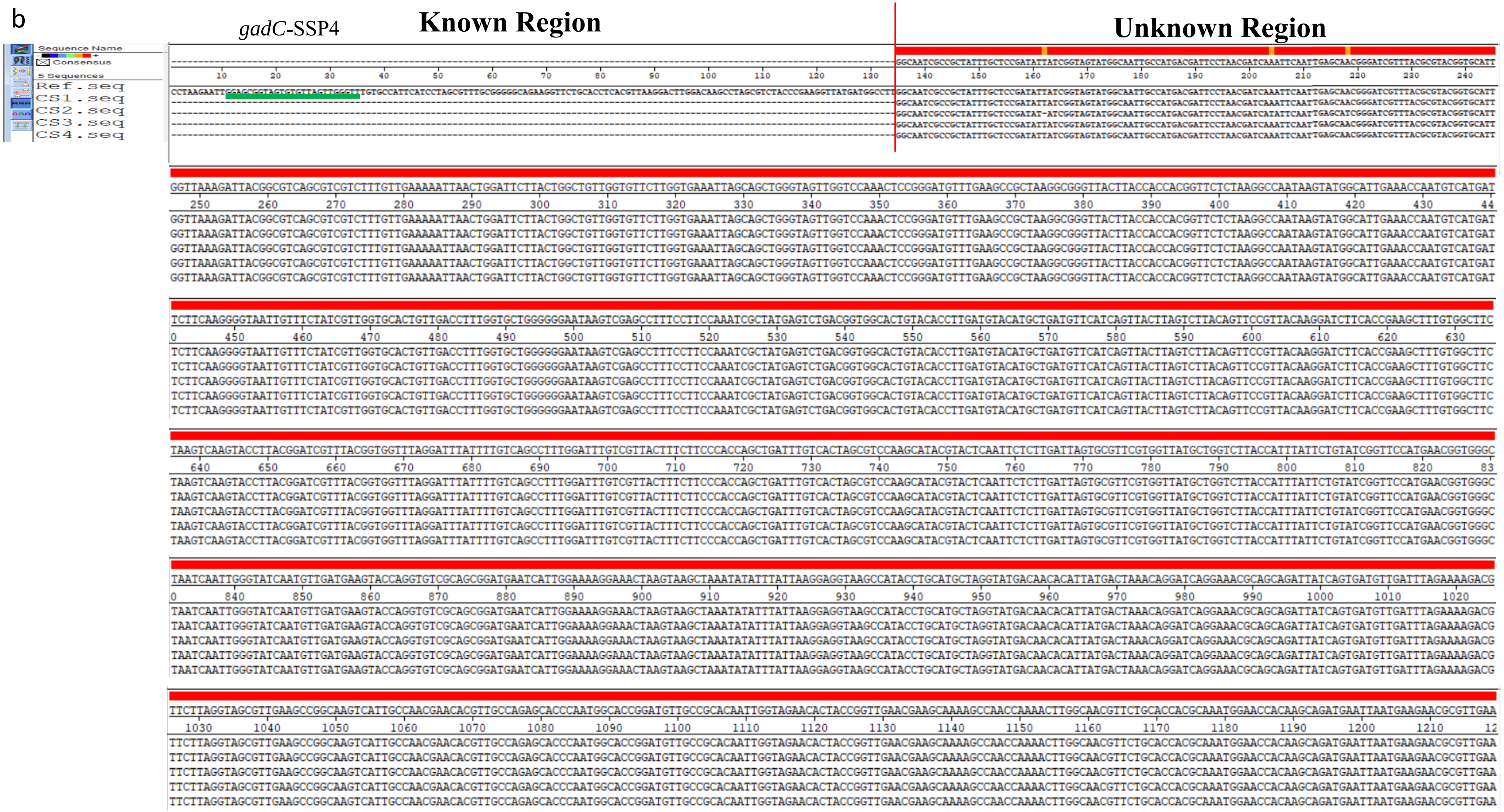
gadC-SSP4
Known Region
Unknown Region
Sequence Name
Consensus
5 Sequences
Ref.seq
CS1.seq
CS2.seq
CS3.seq
CS4.seq

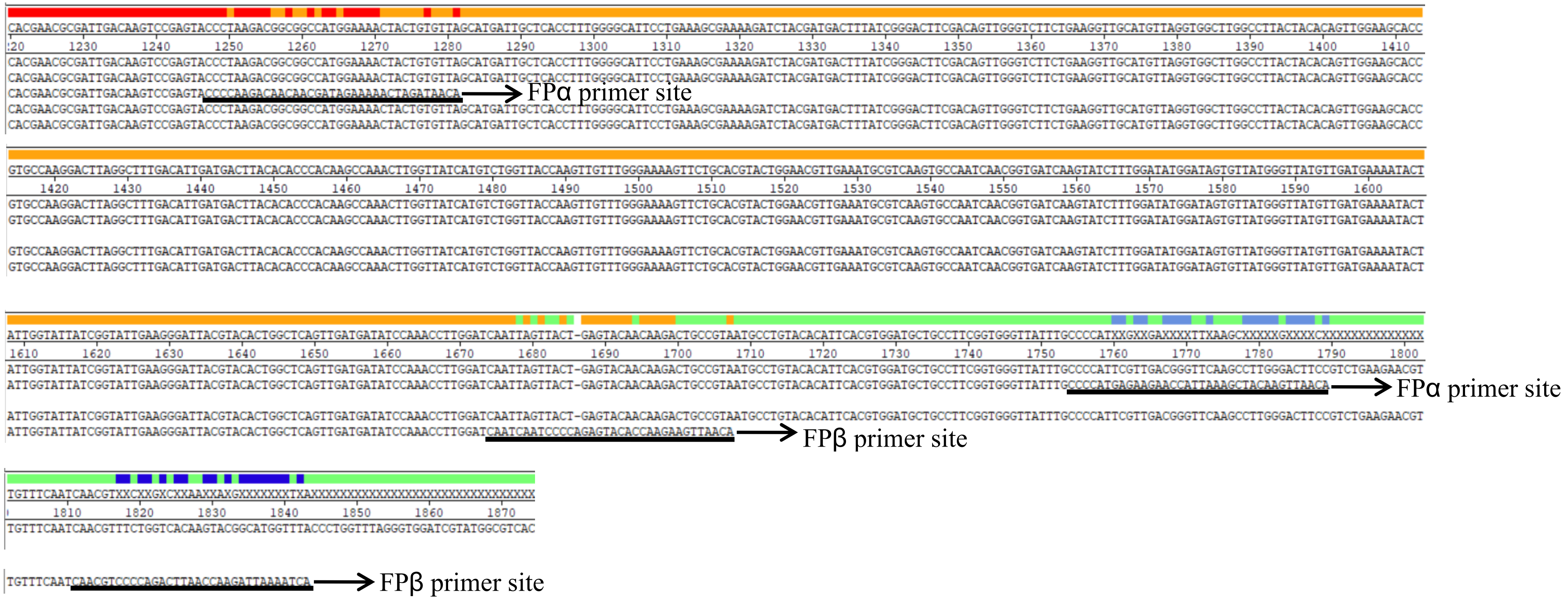
FPα primer site
FPα primer site
FPβ primer site
FPβ primer site

c

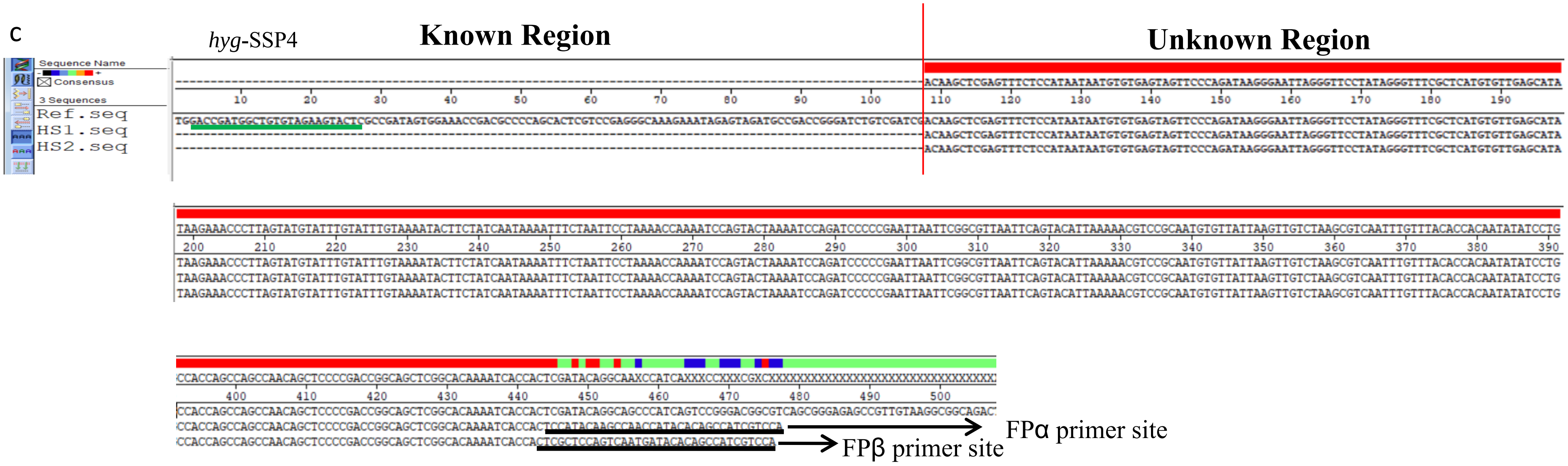

Figure S1 Alignment results of the walked products. a: walking for *gadR*; b: walking for *gadC*; and c: walking for *hyg*. The distances between SSP4 primers and unknown regions of *gadR*, *gadC,* and *hyg* are 71, 99, and 76 bp, respectively. The walked regions are as long as the expected lengths from gel electrophoresis. The names of sequences are shown in the left sides of alignment. Ref refers to the reference sequence; the walked sequences, RS1-RS2, CS1-CS4, and HS1-HS2, correspond to those given in Figure 2.
